# Kinetic nanofriction: a mechanism transition from quasi-continuous to ballistic-like Brownian regime

**DOI:** 10.1186/1556-276X-7-148

**Published:** 2012-02-21

**Authors:** Mehdi Jafary-Zadeh, Chilla Damodara Reddy, Viacheslav Sorkin, Yong-Wei Zhang

**Affiliations:** 1 Department of Materials Science and Engineering, National University of Singapore, 10 Kent Ridge Crescent, Singapore, 119260, Singapore; 2 Institute of High Performance Computing, A*STAR, 1 Fusionopolis Way, #16-16 Connexis North Tower, Singapore, 138632, Singapore

## Abstract

Surface diffusion of mobile adsorbates is not only the key to control the rate of dynamical processes on solid surfaces, e.g. epitaxial growth, but also of fundamental importance for recent technological applications, such as nanoscale electro-mechanical, tribological, and surface probing devices. Though several possible regimes of surface diffusion have been suggested, the nanoscale surface Brownian motion, especially in the technologically important low friction regimes, remains largely unexplored. Using molecular dynamics simulations, we show for the first time, that a C_60 _admolecule on a graphene substrate exhibits two distinct regimes of nanoscale Brownian motion: a quasi-continuous and a ballistic-like. A crossover between these two regimes is realized by changing the temperature of the system. We reveal that the underlying physical origin for this crossover is a mechanism transition of kinetic nanofriction arising from distinctive ways of interaction between the admolecule and the graphene substrate in these two regimes due to the temperature change. Our findings provide insight into surface mass transport and kinetic friction control at the nanoscale.

## Introduction

Atoms, molecules, and nanoparticles are the basic building blocks for many applications in nanotribology and nanomachines including nano-electro-mechanical systems [1-5]. When a bottom-up approach is used, one often has to manipulate these blocks through positioning, packing, and moving them on a surface. Meanwhile, at finite temperature, a building block on a surface may undergo thermally-driven diffusive motion [6], in which it interacts with its surrounding atoms and experiences kinetic friction. Therefore, there is an intrinsic connection between kinetic friction and surface diffusion at the atomic scale, which has recently attracted considerable attention [7,8].

Due to the scientific and technological importance of surface diffusion, a great deal of effort has been devoted to understand the microscopic mechanisms by which adsorbates move on a surface [9]. In systems with strong potential energy barriers and at low temperature, surface diffusion occurs through a series of uncorrelated random jumps between neighboring adsorption sites as described by transition-state theory; while at extremely high temperatures, a crossover from the thermal activated jump regime to the high-temperature Brownian motion regime was theoretically described [10]. The Langevin equation (LE) of motion for an isolated adsorbate is a remarkably successful model of surface diffusion. It characterizes diffusion by two phenomenological parameters: the strength of the potential energy barrier (*E_a_*), and the kinetic friction coefficient (*η*), which indicates the rate at which energy is transferred between the adsorbate and the surface. Supposing independency between *E_a _*and *η*, solutions of the LE has found out four distinct regimes of surface diffusion [9,11]:

*• Regime I (single jumps)*, where the *E_a _*is strong (comparing to the thermal energy (*k_B_T*) of the adparticle), and the *η *is high, the adsorbate mainly resides inside local minima of the potential energy surface (PES). This adsorbate moves by hopping from one minimum to a neighboring minimum. The diffusion rate tends to follow Arrhenius behavior and is well described by the transition-state theory.

*• Regime II (multiple jumps)*, where the *E*_*a *_is strong, and the *η *is low, hopping with long jumps is the dominant feature. The adsorbate may hop from one minimum to a distant local minimum, flying over sites. In the limit of extremely low friction, the microscopic motion is stick-slip, and the trajectory might be characterized by Lévy flight [12].

*• Regime III (quasi-continuous Brownian motion)*, where the *E_a _*is weak, and the *η *is high, the adsorbate moves continuously without being confined to a single local minimum of the PES. In this case, the adsorbate motion is similar to that of a Brownian particle in a high-friction liquid [9].

*• Regime IV (ballistic-like Brownian motion)*, where the *E_a _*is weak, and the *η *is low. The adsorbate moves continuously and travels in linear trajectories at the picosecond time scale, which resembles the directional motion of a projectile. In this case, the adsorbate motion is similar to that of a Brownian particle in a low-friction liquid.

Most of the experimental observations of surface diffusion (especially in the chemisorbed systems with high energy barriers) have been classically described and characterized by the transition-state theory and the single-hop model [13], though at elevated temperatures, multiple jumps are also observed. Characteristics of extremely long jumps (Lévy flight) were observed in systems like gold-cluster/graphite [12] and graphene-flake/graphene [14]. Observation of adsorbates undergoing Brownian motion is relatively rare, even though this behavior is theoretically expected at extremely high temperatures [10]. The first observation of high-friction Brownian motion (Regime III) was in the benzene/graphite system [7]. Nevertheless, to the best of our knowledge, we are unaware of any definite observation of ballistic-like Brownian motion (Regime IV) in a realistic system.

Despite a great deal of experimental and theoretical effort that has been devoted to the study of surface diffusion [9], many issues associated with surface diffusion in the systems with low energy barriers and/or low friction are still not well understood [15]. Therefore, it is necessary to investigate adsorbate/substrate systems with shallow potential energy surface and low friction so as to discover realistic systems which exhibit nanoscale ballistic-like surface Brownian motion. To this end, we considered the C_60_/graphene system which is important in current nanoscience research [16,17]. Miura *et al. *[18] showed that a C_60 _monolayer between the graphite plates exhibits ultra-low friction. Recently, Neek-Amal *et al. *[19] studied the diffusion of C_60 _on graphene at room temperature and reported a shallow potential energy barrier. Hence, from the scientific point of view, the C_60_/graphene system can be considered as an ideal model system to study basic principles of Brownian motion at the nanoscale, and this system is also a promising candidate to exhibit ballistic-like Brownian motion due to its low energy barriers and low surface friction. From the application point of view, fullerenes have been widely considered as a promising material for superlubricity and nanobearing applications [20,21].

In the present work, we use molecular dynamics (MD) simulations to study the motion of an isolated C_60 _molecule on a graphene substrate, focusing on identifying different surface diffusion regimes, their crossover, and their underlying mechanisms. MD simulation is a standard tool to study surface diffusion and have reached a level of accuracy that can often be compared with experimental results [22]. Perhaps more importantly, MD simulations can often be used in scenarios where cannot be reached by experimental techniques. Here, we show, for the first time, that the C_60_/graphene system not only exhibits both Regimes III and IV, but also reveals a crossover between them by simply increasing the temperature of the system.

## Method and model

**Figure 1:**
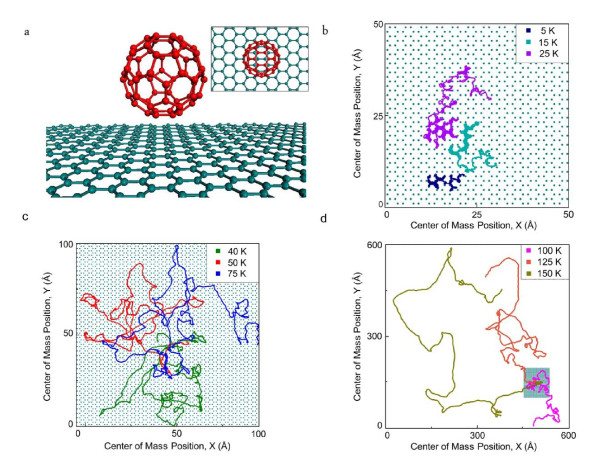
**MD simulations of surface diffusion in C_60_/graphene system**. (**a) **The side view of a C_60 _molecule above a graphene sheet. The inset shows the top view. **(b-d) **Four nanosecond trajectories of C_60 _at **(b) **ultra-low temperature regime. Single hopping mechanism is dominant at 5 K, which gradually turns to multiple jumps up to 25 K. Even at 15 and 25 K, some signs of quasi-continuous motion are observable. (**c**) Low temperature regime, which shows quasi-continuous Brownian motion (Regime III). (**d) **High temperature regime, which shows ballistic-like Brownian motion (Regime IV).

Our computational model, which consists of a single C_60 _molecule on top of a graphene sheet, is illustrated in Figure 1a. The trajectories, energetics, and dynamics calculations are performed in the temperature range of 5 to 200 K.

### MD method and interatomic potential

The C_60_/graphene system was simulated using the large-scale atomic/molecular massively parallel simulator (LAMMPS) code [23] and the adaptive intermolecular reactive empirical bond order AIREBO potential [24]. The AIREBO is one of the most successful potentials applied to model both chemical reactions and intermolecular interactions in condensed-phase hydrocarbon systems, including graphene. The AIREBO potential can provide accurate results for the chemical and mechanical properties of variety of graphene configurations [25,26]. It consists of three terms:

(1) $$E=\frac{1}{2}\underset{i}{\sum }\underset{j\neq i}{\sum }\left[E_{ij}^{REBO}+E_{ij}^{LJ}+\underset{k\neq i,j}{\sum }\underset{l\neq i,j,k}{\sum }E_{kijl}^{TORSION}\right]$$

The first term, a slightly modified version of reactive empirical bond order (REBO) [24], is capable to handle short-range interactions (distance between atoms, *r *< 2 Å) as well as 3-body and 4-body interactions with nearest neighbor atoms in hydrocarbon systems. The second term takes into account the long-range interactions (2 <*r *< cutoff) using the standard Lennard-Jones potential with a cutoff of 12 Å. The third term is an explicit 4-body potential that describes various dihedral angles in hydrocarbon system. It is noteworthy that the REBO potential itself is an application of the Tersoff potential [27,28] in hydrocarbon systems.

In our simulations, the graphene substrate has the dimensions of 100 by 100 Å, consisting of about 3,770 atoms (see Figure 1a). Periodic boundary conditions were applied along the in-plane directions.

### Calculation model and data analysis methods

A C_60 _molecule was positioned on the top of the graphene layer in such a way that one of its hexagon faces was parallel to the graphene. The edges of this hexagon were parallel to the edges of the graphene hexagons, and later on, this configuration is referred as 'Hex.-In Phase'.

In order to study the dynamics of the system, the microcanonical ensemble was selected. The simulations were performed in the temperature range of 5 to 200 K.

At the beginning of each simulation, energy minimization was performed to relax the atomic positions of the system. The Polak-Ribiere version of the conjugate gradient method implemented in the LAMMPS code was used for the energy minimization. After energy minimization, the velocities of the C_60 _molecule and the graphene atoms were assigned following Maxwell-Boltzmann distribution at the desired temperature.

The time step of the Verlet integration algorithm was chosen as 1 fs. Each trajectory calculation was proceeded by a thermal equilibration of 50,000 integration steps (50 ps), followed by a run for 10 ns to extract data for diffusion and friction analysis.

The diffusion coefficients of C_60 _were calculated according to the Einstein's formula using the mean quadratic displacement of its center of mass:

(2) $$D={\text{lim}}_{t\to \infty }\frac{<{\left[X_{CM}\left(t-t_{0}\right)-X_{CM}\left(t_{0}\right)\right]}^{2}{>}_{Nt}}{4t}$$

where **< >*_Nt _***is the ensemble or time averaging over the trajectories, and *X_CM _*is the two-dimensional position vector of the C_60 _center of mass, *t*_*0 *_is the time origin, and *t *is the elapsed time from the time origin.

The surface diffusion process can be described by the linear response theory in which the diffusion coefficient *D *is a function of system temperature and friction coefficient *η*. Following to the Einstein's theory of Brownian motion:

(3) $$D=\frac{k_{B}T}{m\eta }$$

where *m *is the mass of C_60_, we can calculate the friction coefficient of the C_60_/graphene system over the temperature range from 25 to 200 K.

## Results and discussion

**Figure 2:**
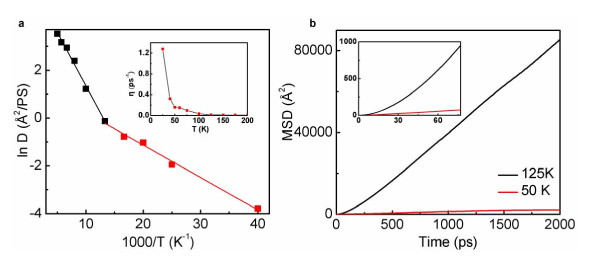
**Effect of temperature on the diffusion coefficient and kinetic friction of the C_60_/graphene system**. (**a**) The Arrhenius analysis of the surface diffusion coefficient, D, of the C_60 _indicates the existence of two diffusive regimes with a crossover around 75 K. The inset of (**a**) illustrates that the friction coefficient decreases from 1.3 ps^-1 ^to a value in the order of 0.01 ps^-1 ^when the temperature is increased from 25 to 200 K. (**b**) Mean square displacement (MSD) of C_60 _motion as a function of time at 50 and 125 K. Note that at 50 K, the MSD grows linearly with time, consistent with quasi-continuous Brownian motion. At 150 K, the MSD is initially parabolic for time shorter than 1/*η*, consistent with ballistic-like Brownian motion.

**Figure 3:**
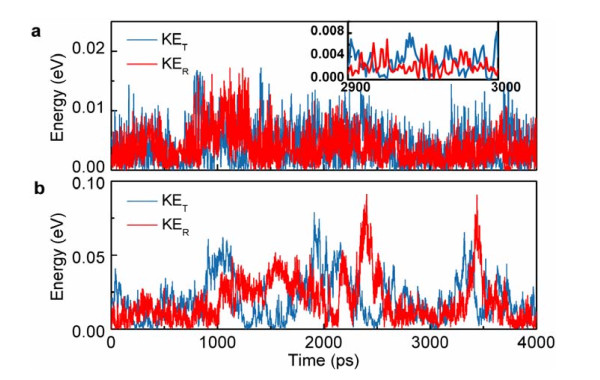
**Conversion between the translational and rotational kinetic energies of C_60 _during surface diffusion on the graphene**. The temperatures are (**a**) T = 50 K, and (**b**) T = 200 K. The inset of **(a) **shows the interplay between the translational and rotational energies as a function of time at T = 50 K with a higher resolution.

The trajectories of the center of mass of the C_60 _molecule are plotted in Figure 1 for: (b) ultra-low, (c) low, and (d) high temperatures. According to Figure 1b, the single jump mechanism is dominant at the very low temperature of 5 K. With further increasing temperature, our simulations show that multiple jumps gradually dominate. At about 25 K, multiple jumps are dominant, although quasi-continuous motion is also present. Hence, up to 25 K, C_60 _on graphene primarily moves through the hopping mechanism. At temperatures above 25 K, however, the trajectories suggest that C_60 _on graphene no longer undergoes hopping, rather it moves continuously. Qualitatively, as it can be seen in Figure 1c, at temperatures of 40, 50, and 75 K, the trajectories of the C_60 _molecule are consistent with quasi-continuous Brownian motion (Regime III), similar to that observed in the benzene/graphite system [7]. When the temperature is increased above 75 K, the trajectories follow a ballistic-like Brownian motion (Figure 1d). To our knowledge, no realistic system was reported previously to exhibit this regime (Regime IV), and a crossover between Regimes III to IV. We support our above statements through quantitative studies of the time-dependence of mean square displacement (MSD) of the C_60 _center of mass, diffusion coefficient, *D*, and the kinetic friction coefficient, *η*. The diffusion coefficient, *D*, is calculated using the best linear fit of the MSD at different temperatures. Einstein's formula for diffusive Brownian motion, *D = k_B_T/(mη)*, is used to obtain the friction coefficient, *η*, where *m *is the mass of C_60_. The variation of diffusion coefficient *D *with temperature is shown in Figure 2a. From this plot, *D *appears to follow Arrhenius behavior with two different regimes, with a crossover in behavior occurring at about 75 K. The measured activation energies are approximately 11 and 36 meVfor the low and high temperature regimes, respectively. The inset of Figure 2a shows the calculated kinetic friction coefficient of the C_60_/graphene system as a function of temperature in the range of 25 to 200 K. It can be seen that as the temperature increases, the friction coefficient *η *decreases drastically from low temperatures to high temperatures. This also indicates that the C_60 _molecule experiences ultra-low kinetic friction at elevated temperatures. This analysis suggests that the kinetic friction coefficient, *η*, is strongly dependent on temperature in C_60_/graphene system. The transition between Regimes III and IV can also be identified by investigating the behavior of MSD of the C_60 _center of mass. Figure 2b shows the MSD curves as a function of time for low (50 K) and high (125 K) temperatures, which correspond to Regime III and IV, respectively. It is clear that at high temperature (125 K), the MSD curve starts off parabolic and accommodates on a straight line after a characteristic time of 1/*η*, which matches the characteristics of Regime IV [9,11]. On the other hand, at 50 K, the parabolic part is not observable, and the MSD curve is readily rectilinear, indicating Regime III [9,11]. The inset of Figure 3b shows the above MSD curves at the short time scale of 77 ps, corresponding to 1/*η *at 125 K, which clearly illustrates the characteristics of these two regimes of Brownian motion [9,11].

Because of the importance of rotational degrees of freedom (DOF) in this system, it is not possible to completely understand the dynamics of the diffusing C_60 _using only the Langevin model. To this end, we examine the interplay between translational and rotational kinetic energies of C_60 _molecule during its motion on the surface. Figure 3a, b shows the distinct energy conversion patterns between rotational and translational modes of C_60 _motion at 50 (in Regime III) and 200 K (in Regime IV), respectively. It can be seen that in Regime III, that is, at the low temperature regime, the energy transfer occurs with a higher frequency comparing to that in Regime IV, that is, the high temperature regime. This pattern suggests that in Regime III, the energy corrugation of the surface (corresponding to the PES) plays an important role in the 'push-pull' of the energy between translational and rotational DOF, and the anti-correlation between these DOF occurs with a higher frequency (see Figure 3a). In contrast, in Regime IV, the overall kinetic energy of the C_60 _is high compared to the shallow PES, and the C_60 _receives extra kinetic energy from the high energy thermally excited graphene atoms in the form of instant kicks. In this regime, the role of the PES is negligible in the 'push-pull' of the energy between translational and rotational DOF. When the high speed C_60 _moves over the graphene surface, it occasionally collides with the surface thermal corrugations. Due to such collisions, the energy is exchanged between the C_60 _and the graphene, as well as between the C_60 _translational and rotational DOF. Generally, such collisions do not lie on the C_60 _center of mass and, thus, create rotational torques. As a result, translational energy is converted into rotational energy. On the other hand, rotating C_60 _may also hit another thermal bump of the surface and pull kinetic energy back into the translation mode. This process is repeated during motion and exhibits as a clear anti-correlation between translational and rotational kinetic energies of the C_60 _(see Figure 3b). The anti-correlation between translational and rotational DOF at high temperatures resembles the 'ballistic nanofriction' process recently described by Guerra *et al*. in the gold-cluster/graphite system [8]. The mechanism of ballistic nanofriction in their work appears to be similar to the mechanism of motion in Regime IV reported here in two ways: first, it also exhibits a clear anti-correlation between rotational and translational kinetic energies; second, their damping mechanism is governed by the thermal corrugation, and not the potential corrugation of the substrate. Nevertheless, there is a fundamental difference between our and their work. In their work, the ballistic regime was achieved by applying a large instantaneous external force to the gold cluster to generate an initial kick. Hence, the gold particle is not in thermal equilibrium, and the linear-response theory and the Einstein's theory of Brownian motion are no longer applicable to their motion.

In the present work, the temperature is limited to 200 K. We have performed simulations with temperatures above 200 K. At these higher temperatures, the C_60 _molecule receives stronger instant kicks from the graphene through thermal fluctuations. Consequently, the C_60 _can break the potential energy barrier, causing desorption of the C_60 _from the graphene.

**Figure 4:**
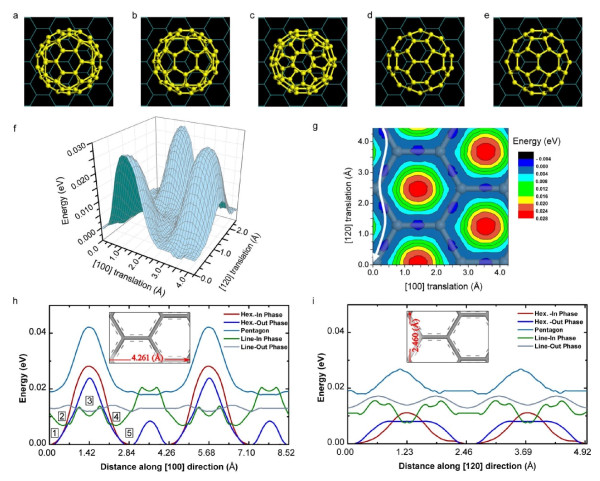
**The effect of rotational degrees of freedom of C_60_ on the potential energy surface (PES). **(**a-e**) Various configurations are used to examine the PES profiles. These configurations are named as: (**a**) Hex.-In Phase; (**b**) Hex.-Out Phase; (**c**) Pentagon; (**d**) Line-In Phase; and (**e**) Line-Out Phase. (**f**) Three-dimensional PES for the Hex.-In Phase configuration. (**g**) Contour plot representation of the PES in (**f**). The white arrow in (**g**) indicates the diffusion path with the lowest energy barrier. The arrangement of graphene atoms and their bonds is also illustrated in this figure. (**h**) and (**i**) show the PES profiles for the C_60_ with different facets, as shown in (**a-e**), during its translation along the [100] and [120] crystallographic directions of graphene, respectively.

Since the PES plays an important role in the characteristics of diffusive and quasi-continuous motion of C_60 _on graphene (Regime III), we examine the PES of C_60_/graphene system by including the effects of both the facets and finite size of C_60_. Figure 4f shows the PES calculated for the Hex.-In Phase configuration of the C_60 _on graphene (see Figure 4a). According to Figure 4f, the magnitude of PES in C_60_/graphene system is in the order of a few meV, which is reasonable in a physisorbed system. Such a 'flat PES', in the order of a few meV, has been also reported recently in the physisorbed benzene/graphite system [7].

The contour plot of the energy surface of Figure 4f is presented in Figure 4g, in which a path (indicated by the white arrow) parallel to the [120] crystallographic direction of the graphene is illustrated. This path indicates a smooth diffusive pathway with a negligible energy barrier of approximately 4 meV. Therefore, one could expect that the trajectories of the C_60 _molecule must be confined in this minimum energy path. However, this is not the case in the temperature range studied in the current work. According to Figure 4h, i, when a C_60 _molecule faces an energy barrier, the molecule can overcome it by rotating to another configuration with an even lower barrier. We illustrate such scenario using Figure 4h: the C_60 _in the Hex.-In Phase orientation may move from point 1 to point 5 along the [100] direction, where it has to overcome an energy barrier of about 0.024 eV. However, at point 2, it can partially tilt to the Line-In Phase orientation (see Figure 4d) and move to point 3 and then to point 4 by crossing a lower energy barrier. After passing point 4, the C_60 _can tilt back to the Hex.-In Phase orientation and continue its way along [100] direction to point 5. Energetically, this whole process is more favorable. Therefore, we conclude that the rotational degrees of freedom of C_60 _together with its faceted shape offer various possible paths on the graphene substrate with low energy barriers. Consequently, there is no preferable diffusion path for C_60 _on graphene in Regime III. It should be noted that chemical modification of graphene, which is widely used to control the electronic properties of graphene, may have significant effects on the PES of the C_60_/graphene system and cause a drastic change in the diffusive behavior of the C_60 _molecule.

## Conclusions

The thermally-induced motion of C_60 _on the graphene substrate with a shallow potential energy surface was investigated. We found that the C_60_/graphene system exhibits two distinct regimes of nanoscale surface Brownian motion. For the first one, the C_60 _molecule exhibits a quasi-continuous Brownian motion (Regime III) in the temperature range of 25-75 K. For the second one, the C_60 _molecule follows a ballistic-like Brownian motion (Regime IV) at temperatures above 75 K. Moreover, these two regimes of Brownian motion imply the existence of two distinct mechanisms of nanoscale kinetic friction, which are responsible for the exchange of energy between the C_60 _molecule and the graphene substrate. In Regime III, the PES and the facets of C60 molecule play a dominant role in the exchange of energy between C_60 _and the substrate. In contrast, the thermal corrugation of the graphene plays a dominant role in Regime IV. The crossover between these two regimes arises from the change in the system temperature. Since there is an intrinsic connection between surface diffusion and kinetic friction at the atomic scale, the present findings not only provide insights into controlling the surface mass transport and nanofriction, but also guidelines for experimentalists to observe and characterize the intriguing diffusive regimes in the C_60_/graphene system and to explore for new materials for nanoscale electro-mechanical applications.

## Competing interests

The authors declare that they have no competing interests.

## Authors' contributions

All authors had equal contribution to this manuscript, and they read and approved the final version.
